# A Model System to Explore the Detection Limits of Antibody-Based Immuno-SPECT Imaging of Exclusively Intranuclear Epitopes

**DOI:** 10.2967/jnumed.120.251173

**Published:** 2021-11

**Authors:** Mathew Veal, Gemma Dias, Veerle Kersemans, Deborah Sneddon, Stephen Faulkner, Bart Cornelissen

**Affiliations:** 1 Medical Research Council Oxford Institute for Radiation Oncology, Department of Oncology, University of Oxford, Oxford, United Kingdom; and; 2 Department of Chemistry, University of Oxford, Oxford, United Kingdom

**Keywords:** molecular imaging, nuclear targeting, PET, SPECT, cell-penetrating peptide, TAT

## Abstract

Imaging of intranuclear epitopes using antibodies tagged to cell-penetrating peptides has great potential given its versatility, specificity, and sensitivity. However, this process is technically challenging because of the location of the target. Previous research has demonstrated a variety of intranuclear epitopes that can be targeted with antibody-based radioimmunoconjugates. Here, we developed a controlled-expression model of nucleus-localized green fluorescent protein (GFP) to interrogate the technical limitations of intranuclear SPECT using radioimmunoconjugates, notably the lower target-abundance detection threshold. **Methods:** We stably transfected the lung adenocarcinoma cell line H1299 with an enhanced GFP (EGFP)–tagged histone 2B (H2B) and generated 4 cell lines expressing increasing levels of GFP. EGFP levels were quantified using Western blot, flow cytometry, and enzyme-linked immunosorbent assay. An anti-GFP antibody (GFP-G1) was modified using dibenzocyclooctyne-N_3_–based strain-promoted azide–alkyne cycloaddition with the cell-penetrating peptide TAT (GRKKRRQRRRPPQGYG), which also includes a nuclear localization sequence, and the metal ion chelator N_3_-Bn-diethylenetriamine pentaacetate (DTPA) to allow radiolabeling with ^111^In. Cell uptake of ^111^In-GFP-G1-TAT was evaluated across 5 cell clones expressing different levels of H2B-EGFP in vitro. Tumor uptake in xenograft-bearing mice was quantified to determine the smallest amount of target epitope that could be detected using ^111^In-GFP-G1-TAT. **Results:** We generated 4 H1299 cell clones expressing different levels of H2B-EGFP (0–1 million copies per cell, including wild-type H1299 cells). GFP-G1 monoclonal antibody was produced and purified in house, and selective binding to H2B-EGFP was confirmed. The affinity (dissociation constant) of GFP-G1 was determined as 9.1 ± 3.0 nM. GFP-G1 was conjugated to TAT and DTPA. ^111^In-GFP-G1-TAT uptake in H2B-EGFP–expressing cell clones correlated linearly with H2B-EGFP expression (*P* < 0.001). In vivo xenograft studies demonstrated that ^111^In-GFP-G1-TAT uptake in tumor tissue correlated linearly with expression of H2B-EGFP (*P* = 0.004) and suggested a lower target-abundance detection threshold of approximately 240,000 copies per cell. **Conclusion:** Here, we present a proof-of-concept demonstration that antibody-based imaging of intranuclear targets is capable both of detecting the presence of an epitope of interest with a copy number above 240,000 copies per cell and of determining differences in expression level above this threshold.

## Visual Abstract

**Figure:**
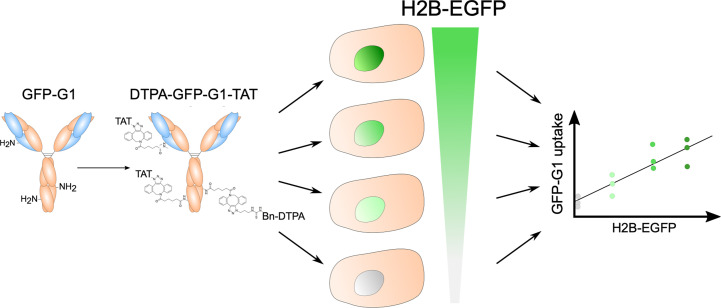

Molecular imaging enables noninvasive characterization of biochemical features at a molecular level, performed on anything from a living cell to an entire organism (1). In parallel with other branches of personalized precision medicine, molecular imaging has become a rapidly expanding field of research, providing applications including early diagnostic tools, patient stratification, therapy guidance, and posttherapy evaluation. Nuclear imaging by PET or SPECT using radiolabeled modified antibodies, or radioimmunoconjugates, has already shown great promise in cancer imaging because of the specificity, versatility, and dependable pharmacokinetics unique to monoclonal antibodies (2). Although most research has focused on the development of monoclonal antibodies targeting extracellular epitopes on cancer cell membranes, the extracellular matrix, or epitopes shed into the interstitial space, approximately 30% of cellular proteins are localized within the nucleus, orchestrating a myriad of physiologically and pathologically relevant processes (3). The opportunity to successfully target intranuclear epitopes would significantly expand the potential applications of molecular imaging.

Without modification, antibodies are unable to cross cellular membranes because of their size (∼150,000 Da) and hydrophilicity. However, these barriers can be overcome using cell-penetrating peptides (CPPs) (4). CPPs are short-length peptides (<30 residues) that have the capacity to translocate across cellular membranes (5). Since the initial discovery of the membrane transduction capacities of the HIV-derived TAT (GRKKRRQRRRPPQGYG) peptide and *Drosophila* antennapedia homeodomain protein–derived peptide (6–8), over 1,800 CPPs have been described (9). In addition, many CPPs have been experimentally validated in vitro and in vivo to facilitate the translocation of bioactive molecular cargoes of various sizes, up to 540,000 kDa, across cellular membranes, with limited toxicity (10).

Apart from serving as a CPP, the TAT peptide also contains a noncanonic nuclear localization sequence enabling nuclear translocation of its cargo. Previous research from our group and others has demonstrated that TAT-peptide–conjugated antibodies (IgG-TAT) can be used to image several intranuclear targets, including p21 (11), p27 (12), and the phosphorylated histone protein H2AX using both PET and SPECT (13–17). This proof-of-concept work has provided a tantalizing glimpse into the potential of IgG-TAT–based PET or SPECT imaging of intranuclear targets.

Given the unparalleled adaptability of antibodies, the range of possible imaging applications using intranuclear IgG-TAT imaging probes is substantial. However, as with all imaging modalities, fundamental limitations in sensitivity are to be expected. Therefore, determining the minimum target-epitope copy number required for antibody-based PET or SPECT imaging would be highly beneficial when novel protein markers are considered as potential targets. In vivo imaging using antibody fragments has been demonstrated with extracellular epitopes with copy numbers as low as 25,000 and 8,000 copies per cell (18,19). Intracellular epitope detection limits would be expected to be significantly higher, but a quantitative description of this limit has not yet been explored.

To determine the lower threshold of target abundance required for successful intranuclear imaging using radioimmunoconjugates, we developed a model system expressing different levels of a well-characterized, stably expressed, nucleus-localized target protein construct, histone 2B (H2B)–tagged enhanced green fluorescent protein (EGFP). This strategy removes several experimental factors that could undesirably alter target abundance, such as the potential for the probe to influence target functionality and the innate variability and temporal changeability in dosages and responses when imaging-target epitopes are induced by exogenous treatment. Removing these factors allowed for a more rigorous evaluation of the technical capabilities of antibody-based intranuclear imaging.

## MATERIALS AND METHODS

### Cell Culture

Human lung adenocarcinoma H1299 cells (ATCC) were cultured in Dulbecco modified Eagle medium (Sigma), supplemented with 10% fetal bovine serum (Life Technologies), 2 mM l-glutamine, 100 units/mL penicillin, and 0.1 mg/mL streptomycin and maintained in a 5% CO_2_ humidified atmosphere at 37°C. Cells were tested and authenticated by the providers. The cumulative length of culture was less than 6 mo after retrieval from liquid nitrogen storage. Cells were tested for the absence of *Mycoplasma* at regular intervals. Cells stably transfected with H2B-EGFP plasmid were cultured with 50 µg/mL G418/geneticin to promote stability of transfection. Cells were harvested and passaged as required using trypsin–ethylenediaminetetraacetic acid solution.

### Stable Cell Transfection and Selection

H1299 cells were seeded onto 6-well cell culture plates and allowed to attach overnight to reach 75%–90% confluency. Cells were then transfected with H2B-GFP plasmid (catalog no. 11680; Addgene) (20) using lipofectamine 3000 (Invitrogen) according to manufacturer protocols. After transfection (48 h), stably transfected cells were selected by culturing with 800 µg/mL G418 antibiotic for several passages. Cells that survived initial selection were sorted for EGFP green fluorescence and plated as individual cells using flow-assisted cell sorting (FACSAria III; BD Biosciences) (Supplemental Fig. 1A; supplemental materials are available at http://jnm.snmjournals.org). Individual cell colonies were expanded under G418 selection (200 µg/mL), and H2B-EGFP expression in candidate cell lines was initially assessed qualitatively using immunofluorescence microscopy. After establishing 4 stably transfected cell lines, in addition to untransfected H1299 cells, which were used as a negative control, H2B-EGFP expression was evaluated by Western blot, immunofluorescence microscopy, flow cytometry, and enzyme-linked immunosorbent assay (ELISA). Stability of H2B-EGFP expression in the absence of G418 was determined using flow cytometry (Supplemental Fig. 1B).

### Production and Purification of GFP-G1 Monoclonal Antibody

The GFP-G1 mouse monoclonal antibody and associated hybridoma cell line were obtained from the Developmental Studies Hybridoma Bank (University of Iowa; as deposited by Joshua R. Sanes and Masahito Yamagata) (21). Hybridoma cells were initially cultured from frozen stocks in RPMI medium supplemented with 10% fetal bovine serum (Life Technologies), 2 mM l-glutamine, 100 units/mL penicillin, and 0.1 mg/mL streptomycin in a 5% CO_2_ humidified atmosphere at 37°C. Once they displayed exponential growth rates, hybridoma cells were maintained in CD Hybridoma medium (Thermo Fisher Scientific) before being harvested 14 d after the final passage. Supernatant enriched with GFP-G1 antibody was clarified by centrifugation (1,400*g,* 10 min) and filtered through a 0.2-µm filter using a peristaltic pump before fast protein liquid chromatography purification using a HiTrap Protein G HP antibody purification column (Cytiva). Purified antibody was buffer-exchanged to phosphate-buffered saline (PBS) solution and stored at −20°C. Antibody specificity and affinity (dissociation constant) were characterized using immunofluorescence microscopy and flow cytometry.

### Bioconjugation and Radiolabeling

Bioconjugation of GFP-G1 with CPP TAT and the metal ion chelating agent diethylenetriamine pentaacetate (DTPA) was achieved using strain-promoted azide–alkyne cycloaddition in a non–site-specific manner. GFP-G1 was buffer-exchanged in 0.1 M NaHCO_3_ (pH 8.5), concentrated to 25 µM, and reacted with a 7-fold molar excess of dibenzocyclooctyne (DBCO)-4-sulfo-2,3,5,6-tetrafluorophenyl (Click Chemistry Tools) dissolved in dimethyl sulfoxide at room temperature for 4 h. The antibody–DBCO conjugate was purified and buffer-exchanged to PBS by centrifugal filtration using a Zeba Spin desalting column (7,000 molecular weight cutoff, 0.5 mL; Thermo Scientific). The amount of DBCO tags conjugated to the antibody was determined by absorbance spectroscopy at 280 and 309 nm, with a DBCO-to-antibody ratio of 5:1. The antibody–DBCO conjugate, at approximately 10 µM, was then reacted with a 2-fold molar equivalent relative to DBCO of 3.4 mM N_3_-Bn-DTPA dissolved in PBS plus 20% dimethyl sulfoxide (Supplemental Fig. 2; the supplemental materials provide full synthesis details) and, concurrently, a 0.5-fold molar equivalent (relative to DBCO) of 1 mM azide-tagged CPP TAT-AHA (GRKKRRQRRRPPQGYG-hA(N_3_)) dissolved in PBS (Cambridge Peptides) at 4°C for 12 h. The antibody conjugate was purified and buffer-exchanged to 0.5 M sodium citrate (pH 5.4) to remove unreacted products as previously described. The completion of the conjugation reaction was confirmed by the reduction of ultraviolet absorption spectrometry at 309 nm to background level.

Immunoconjugates were radiolabeled by addition of ^111^InCl_3_ (Perkin Elmer). Aliquots of 150 µg of IgG conjugate (1 mg/mL in 0.5 M sodium citrate, pH 5.4) were reacted with ^111^InCl_3_ (1 MBq/µg of antibody) in 0.5 M sodium citrate buffer, pH 5.4, for 1 h at room temperature. Radiolabeling yield was determined by instant thin-layer chromatography to be greater than 95%. The resulting radiolabeled compound, ^111^In-GFP-G1-TAT, was buffer-exchanged to PBS to remove free ^111^In^3+^ before subsequent studies.

### In Vitro Studies

Aliquots of 8 × 10^4^ cells per well were plated in a 24-well cell culture plate and allowed to adhere for 16 h. Culture medium was removed, and cells were washed with PBS before addition of fresh culture medium containing ^111^In-GFP-G1-TAT (1.5 nM, 1 MBq/µg) and incubation at 37°C for a further 60 min. Supernatant containing unbound radiolabeled compound was collected; cells were washed twice with PBS and then were washed for 5 min on ice in 0.1 M glycine solution, pH 2.5, to remove membrane-bound radiolabeled antibody conjugate. Finally, cells were lysed by addition of 0.1 M NaOH, and cell-associated compound was collected. The amount of ^111^In within each fraction was determined by automated γ-counting (Wizard^2^ 2480; Perkin Elmer), to calculate the percentage of cell-associated ^111^In.

### In Vivo Studies

All animal procedures were performed in accordance with the U.K. Animals (Scientific Procedures) Act of 1986 and with local ethical committee approval. Animals were housed in individually ventilated cages in sex-matched groups of up to 6 per cage in an artificial day–night cycle facility with ad libitum access to food and water. No animals were culled for welfare reasons. Xenografts of H1299 cells, wild-type (WT) or stably transfected with varying levels of H2B-EGFP, were established by subcutaneous inoculation of 10^6^ cells in a 1:1 (v/v) PBS:matrigel mix, on the right flank of female BALB/c *nu/nu* mice (Envigo). After 3–4 wk, tumors reached an average size of 180 mm^3^ and were used for subsequent studies.

^111^In-GFP-G1-TAT (5 MBq/5 µg) in PBS was administered to each mouse via the lateral tail vein. SPECT/CT images were acquired using a VECTor^4^CT scanner (MILabs) at 24 and 72 h after injection. Volume-of-interest analysis on SPECT images was performed using PMOD software (PMOD Technologies). Full experimental details, reconstruction parameters, and acquisition parameters are provided in the supplemental materials (22). After imaging, mice were culled, and samples of blood and other selected organs were collected to determine the percentage of the injected dose per gram of tissue (%ID/g) using a Hidex automatic γ-counter (Hidex Oy). Tumor samples were immediately counted before flash freezing with dry ice and storage at −80°C for future analysis.

### Statistical Analysis

All statistical analysis was performed using Prism, version 8 (GraphPad Software). Linear regression with an extra-sum-of-squares *F* test was used to determine the statistical significance of correlations between estimated regression parameters. Either 1-way ANOVA with a Tukey post hoc test or 2-way ANOVA was used to compare individual group means, as appropriate. All data were collected at least in triplicate and are presented as mean ± 1 SD unless otherwise stated.

## RESULTS

### Stably Transfected H1299/H2B-EGFP Cells Are Generated

**FIGURE 1.**
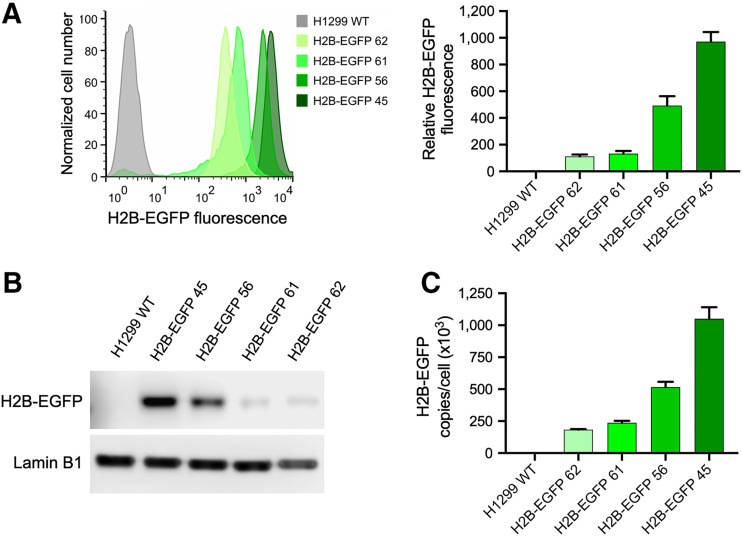
Establishment of H1299 cell lines stably transfected with H2B-EGFP. (A) Relative difference in H2B-EGFP expression across all transfected cell lines, determined by flow cytometry and presented as batch-normalized bar chart. (B) Confirmation of difference in H2B-EGFP expression across transfected cell lines using Western blot. (C) Copy number per cell of H2B-EGFP in each transfected cell line, quantified by ELISA.

**FIGURE 2.**
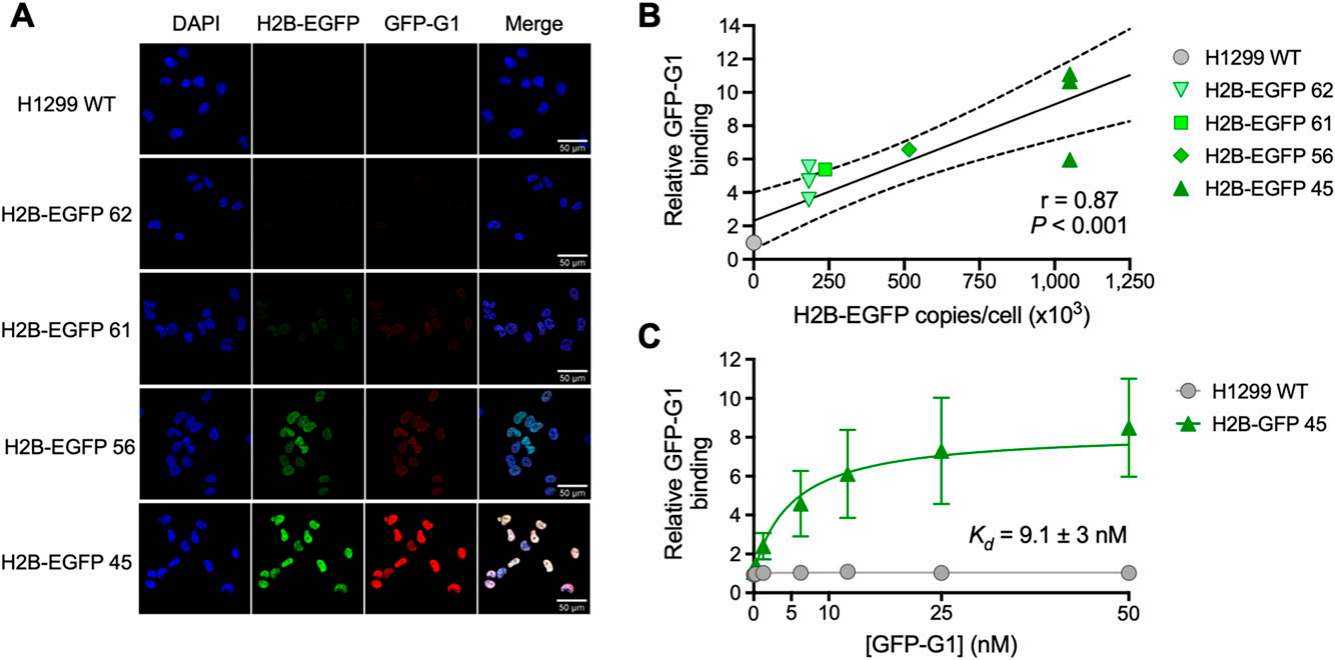
Validation and characterization of GFP-G1 monoclonal antibody. (A) Immunofluorescence microscopy showing colocalization between H2B-EGFP expression and GFP-G1 monoclonal antibody. (B) Confirmation of correlation between increasing H2B-EGFP expression and binding of monoclonal antibody GFP-G1, using flow cytometry. (C) Determination of dissociation constant of GFP-G1 with flow cytometry–based saturation binding assay.

To test the effects of target abundance on imaging ability, we generated a panel of H1299 cell lines that constitutively expressed EGFP tagged at its amino terminus with the histone protein H2B through stable transfection with the mammalian expression plasmid H2B-EGFP. Several colonies of H1299 cells stably expressing H2B-EGFP at various levels were generated, and 4 were selected for future studies, named H2B-EGFP 45, 56, 61, and 62, as well as WT H1299 cells. Expression levels of H2B-EGFP were measured by flow cytometry and Western blot (Figs. 1A and 1B). Expression levels of H2B-EGFP were quantified by ELISA (Fig. 1C) and ranged between 183,000 ± 4,900 and 1,050,000 ± 91,100 copies per cell. These results were corroborated by fluorescence confocal microscopy, which further confirmed exclusively nuclear EGFP expression (Fig. 2A).

### Murine Monoclonal Antibody GFP-G1 Selectively Binds H2B-EGFP

The murine monoclonal anti-GFP antibody GFP-G1 was characterized by flow cytometry with each selected H2B-EGFP transfected cell line along with a H1299 WT control, in fixed and permeabilized cells, confirming earlier results on its selectivity (Figs. 2A and 2B) (23). Fluorescence intensity correlated linearly with H2B-EGFP expression, as measured by ELISA (Fig. 2B, *r* = 0.87, *P* < 0.001). The specificity of GFP-G1 was further supported by immunofluorescence microscopy showing colocalization of H2B-EGFP, GFP-G1, and the DNA stain 4′,6-diamidino-2-phenylindole (Fig. 2A). Immunofluorescence microscopy also demonstrated a correlation between H2B-EGFP expression and GFP-G1 signal intensity. A flow cytometry–based saturation binding assay was used to determine the affinity (dissociation constant) of GFP-G1 for GFP as 9.1 ± 3.0 nM (Fig. 2C).

### In Vitro Uptake of ^111^In-GFP-G1-TAT Correlates with Nuclear GFP Expression

**FIGURE 3.**
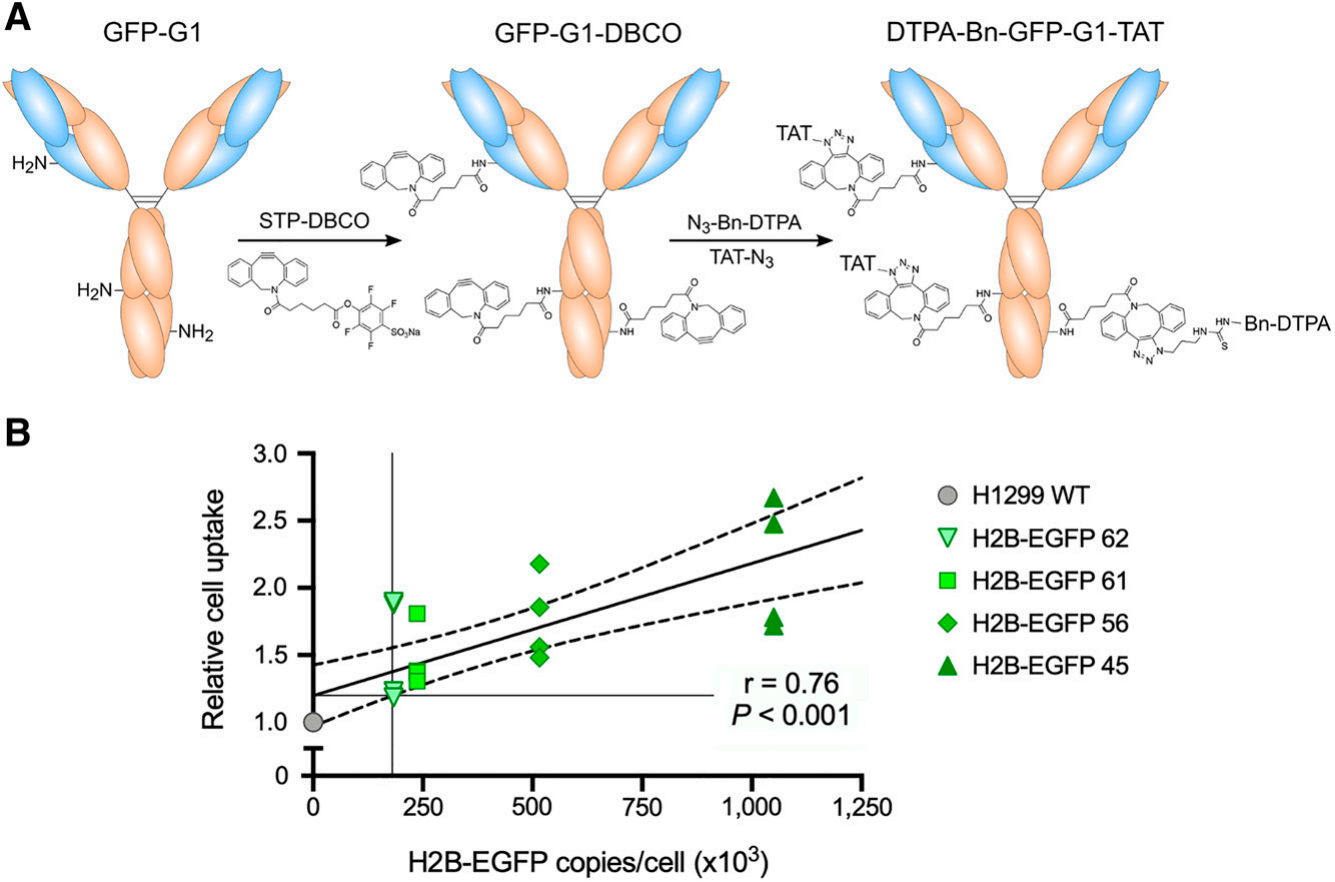
Conjugation and in vitro evaluation of ^111^In-GFP-G1-TAT uptake. (A) Schematic diagram outlaying approach to conjugate monoclonal antibody GFP-G1 with CPP TAT and chelating agent Bn-DTPA using strain-promoted azide–alkyne cycloaddition–based click chemistry. (B) In vitro radiointernalization assay revealing correlation between ^111^In-GFP-G1-TAT uptake and H2B-EGFP expression.

GFP-G1 was conjugated to the CPP TAT and metal ion chelating agent DTPA before radiolabeling with ^111^InCl_3_ (Fig. 3A). In vitro uptake assays showed that ^111^In-GFP-G1-TAT uptake in the panel of 5 cell lines correlated linearly with increasing H2B-EGFP expression (Fig. 3B, *r* = 0.76, *P* < 0.001), suggesting that uptake was the result of specific binding between ^111^In-GFP-G1-TAT and intranuclear H2B-EGFP. In a comparison with a non-TAT modified ^111^In-GFP-G1, using the highest H2B-EGFP–expressing H2B-EGFP 45 cell line, ^111^In-GFP-G1-TAT showed a 6.8-fold increase in cell uptake (Supplemental Fig. 3, *P* = 0.026), demonstrating the requirement of the CPP TAT in the internalization of the imaging probe. Finally, a colony formation assay was used to determine whether the uptake of ^111^In-GFP-G1-TAT and the subsequent radiation dose affected cell viability. In both the H1299 WT and the H2B-GFP 45 cells, there was no significant difference in colony formation among cells exposed to ^111^In-GFP-G1-TAT, cells exposed to ^111^In-GFP-G1 without TAT, and an untreated control group (Supplemental Fig. 4).

### Tumor Uptake of ^111^In-GFP-G1-TAT Correlates with Nuclear GFP Expression and Reveals the Detection Limit of IgG-TAT Architecture

**FIGURE 4.**
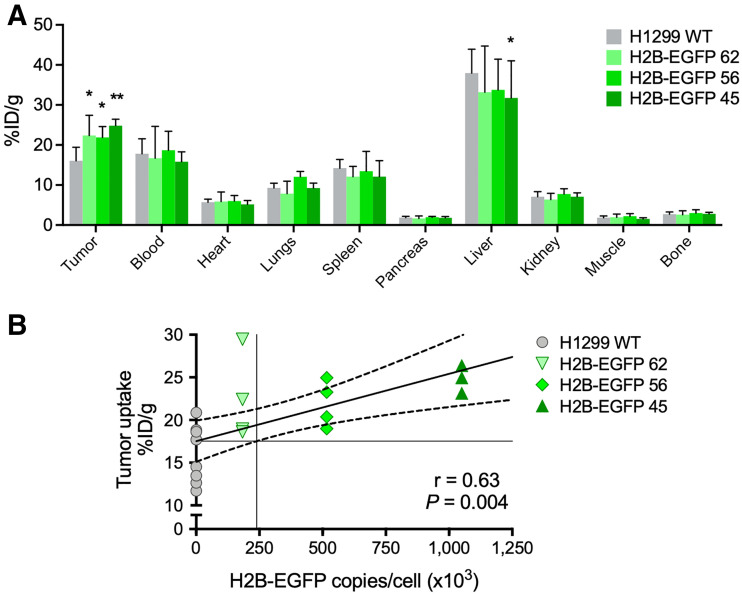
Ex vivo biodistribution and tumor uptake of ^111^In-GFP-G1-TAT. (A) Ex vivo biodistribution showing uptake of ^111^In-GFP-G1-TAT in selected tissues and blood, 72 h after intravenous administration. (B) Tumor uptake of ^111^In-GFP-G1-TAT correlating linearly in vivo with expression of H2B-EGFP. Asterisks denote statistically significant differences in uptake from H1299 WT group. *P < 0.05. **P < 0.01.

^111^In-GFP-G1-TAT could detect H2B-EGFP in a xenograft animal model bearing tumors consisting of H2B-EGFP 45, 56, 62, and H1299 WT cells. Ex vivo biodistribution studies revealed a statistically significant linear correlation between tumor uptake of ^111^In-GFP-G1-TAT and expression of H2B-EGFP (Figs. 4A and 4B, *r* = 0.63, *P* = 0.004), demonstrating that ^111^In-GFP-G1-TAT can quantitatively determine the level of H2B-EGFP expression within tumor xenografts in vivo. Interpolation of the linear regression model suggested a lower H2B-EGFP copy number threshold for detectable uptake of ^111^In-GFP-G1-TAT of approximately 240,000 copies per cell, which was higher than the predicted threshold in vitro (182,000 copies per cell). This was supported by the statistically significant difference in uptake between H1299 WT (16.1 ± 3.4 %ID/g) and H2B-EGFP 45 (24.8 ± 1.6 %ID/g; *P* = 0.010) and H2B-EGFP 56 (21.9 ± 2.7 %ID/g; *P* = 0.033). Interestingly, the uptake of H2B-EGFP 62 (22.4 ± 5.0 %ID/g) was also significantly higher than that of H1299 WT (*P* = 0.018), implying that the detection threshold may even be lower.

**FIGURE 5.**
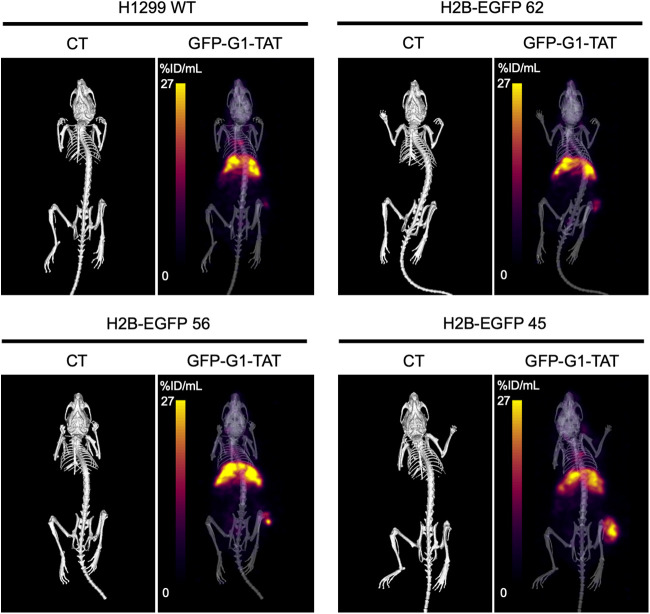
Imaging of ^111^In-GFP-G1-TAT in vivo. Representative CT and SPECT images show distribution of ^111^In-GFP-G1-TAT in mice bearing H2B-EGFP–transfected H1299 cells, 72 h after intravenous administration.

**FIGURE 6.**
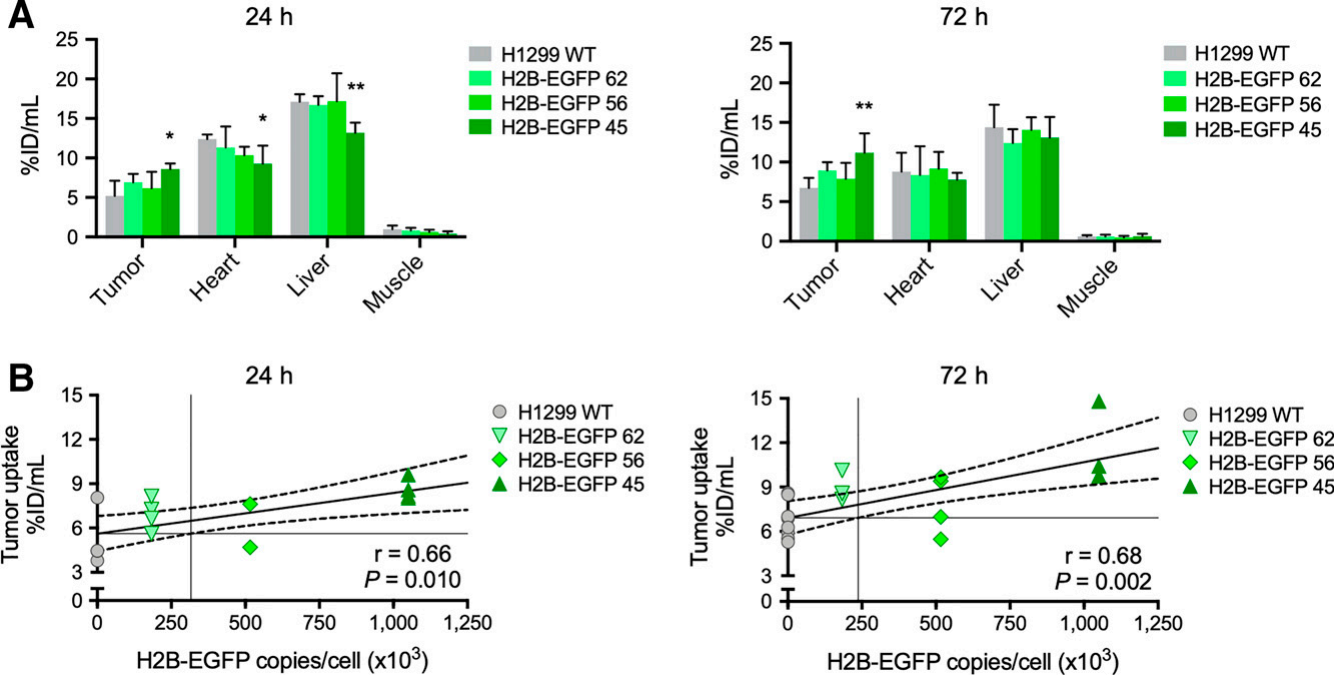
Image quantification analysis of ^111^In-GFP-G1-TAT biodistribution in vivo. (A) Image quantification analysis showing uptake of ^111^In-GFP-G1-TAT in selected tissues, 24 and 72 h after intravenous administration. (B) Correlation of quantified tumor uptake with expression of H2B-EGFP, 24 and 72 h after intravenous administration. Asterisks denote statistically significant differences in uptake from H1299 WT group. *P < 0.05. **P < 0.01. ***P < 0.001.

Uptake of ^111^In-GFP-G1-TAT with increasing expression of H2B-EGFP was also demonstrated visually by SPECT/CT imaging (Fig. 5). Image quantification of uptake again showed a correlation between uptake and expression of H2B-EGFP at both 24 h (*r* = 0.66, *P* = 0.010) and 72 h (*r* = 0.68, *P* = 0.002) after intravenous administration (Figs. 6A and 6B). At 24 h after administration, interpolation of the linear regression model suggested a lower detection threshold of 316,000. However, SPECT images at 72 h after administration suggested a lower detection threshold of 237,000 copies per cell, which reflected the results obtained with the biodistribution study.

## DISCUSSION

Antibody-based or antibody fragment–based immuno-PET or SPECT imaging is a rapidly growing field, with an increasingly broad selection of radioisotopes to use along with a repertoire of highly sensitive and selective antibody-based and antibody fragment–based vector systems that can be optimized for specific applications. The effectiveness of any antibody-based imaging probe can be reduced to 3 central factors (24). The first factor is target abundance, which must be suitably high to allow its detection within the tissue of interest. Second, the targeting component of the radioimmunoconjugate, whether an antibody or antibody fragment, must bind exclusively to the epitope, with high affinity. Finally, the radioimmunoconjugate must exhibit the pharmacokinetic and clearance characteristics in vivo that will allow it to develop a contrast with nontarget tissues. In this study, we used a stable transfection reporter system to investigate the consequence of decreasing target abundance on radioimmunoconjugate detection and imaging capacity.

We chose H2B-EGFP as a model target protein because it is a well-characterized recombinant nucleus-localized protein expression system that is capable of stable transfection (20,25). In vitro assays demonstrated that ^111^In-GFP-G1-TAT uptake correlated with the expression level of H2B-EGFP, with a predicted minimum detection threshold limit of approximately 182,000 copies per cell. Other reports, relying on quantitative mass spectrometry in U2OS and He-La cells, showed that up to a fifth of all nuclear proteins are expressed to this level and thus may be visualized using a TAT-conjugated suitable antibody, although this analysis does not include pharmacologically or pathologically induced expression or overexpression of proteins (26,27). The relatively small increase in ^111^In-GFP-G1-TAT uptake between H1299 WT and H2B-EGFP–expressing cells in vitro was indicative of high levels of nonspecific uptake, possibly as a result of modification with the TAT peptide. However, this system can be used to optimize a multitude of factors to increase specific radioimmunoconjugate uptake, potentially lowering the effective detection threshold and improving the target-abundance resolution of IgG-TAT–based imaging compounds.

The basis of an effective radioimmunoconjugate imaging probe is a highly selective and sensitive antibody or fragment. We selected the GFP-G1 monoclonal antibody because there was a readily available and validated hybridoma cell line (21,23) allowing for production and purification on a scale suitable for bioconjugation and subsequent radioassays. Flow cytometry and immunofluorescence microscopy confirmed that GFP-G1 was highly specific and showed good affinity (dissociation constant, 9.1 ± 3.0 nM), comparable to clinically approved antibodies such as trastuzumab (5.0 nM) (28). However, it should be noted that highly optimized antibodies or antibody-based constructs are capable of affinities in the subpicomolar range (29). An important consideration for future research is whether an antibody with greater affinity could improve the detection threshold and resolution by reducing the signal-to-noise ratio.

The conjugation of functional groups, such as TAT and DTPA, can considerably alter the characteristics of a radioimmunoconjugate. At the cellular level, the addition of TAT facilitates the transport of the radioimmunoconjugate across the cellular membrane. However, because of the highly cationic nature of TAT, TAT-modified radioimmunoconjugates can result in significant increases in nonspecific binding. One of the primary factors that could control the detection threshold and degree of nonspecific uptake is the mechanism of IgG-TAT internalization into cells. CPPs have been shown to facilitate the internalization of wide array of cargo proteins, which is believed to be largely driven by endocytosis under physiologic conditions (30). However, because of the complex array of CPPs and CPP–cargo combinations (31), attempts to establish the principal endocytic mechanisms involved have proved ambiguous, suggesting that CPPs can use distinct endocytic pathways. One major factor governing the efficiency of internalization under physiologic conditions is the molecular mass of the cargo protein cargo size (32). Larger cargo proteins, such as whole antibodies, are internalized at significantly lower efficiencies than small cargo proteins, potentially caused by induction of alternative cell internalization mechanisms (31,33,34).

Another major challenge facing CPP-mediated intranuclear imaging is nonspecific uptake due to endosomal entrapment, resulting in high cellular uptake but poor cytosolic availability and subsequent target engagement. The recent development of cyclic derivatives of TAT and poly-arginine CPPs has demonstrated not only dramatic increases in cell uptake of cargo proteins (35) but also significantly improved cytosolic availability in comparison to the linear CPPs (36). This model system provides an excellent opportunity to investigate the effects of such optimizations for specific cellular uptake of targeted radioimmunoconjugates.

Finally, the choice of chelating agent and radioisotope can also affect the sensitivity and degree of nonspecific tumor uptake. Because the principal objective of this study was to investigate the biodistribution and pharmacokinetic properties of an IgG-TAT radioimmunoconjugate, DTPA was used to chelate ^111^In for simplicity of radiolabeling and to maintain consistency with previous research using TAT-antibody imaging agents. Radiometal-chelate stability is important when using a nucleus-targeted radioimmunoconjugate, so as to prevent nonspecific sequestration of the radiometal and limit subsequent off-target radiation dose, especially when working with an Auger electron–emitting isotope, such as ^111^In (37). Alternative chelating agents—for instance, CHX-A″-DTPA—have greater stability in vivo and therefore could be used to chelate ^111^In (38). For clinical use, PET has several advantages over SPECT, including greater sensitivity, easier scatter correction, and capacity to provide quantitative results (39). Therefore, to increase the potential for clinical translation, future research will be focused on combining this technology with long–half-life PET isotopes, such as ^89^Zr, in conjunction with a suitable chelating agent, such as desferrioxamine B.

In vivo, both biodistribution and quantitative image analysis indicated that the minimum detection threshold was 240,000 copies per cell, which was predictably higher than the threshold obtained from in vitro studies. The reason for the higher observed threshold in vivo may be attributed to increased nonspecific tumor uptake as a result of the enhanced permeability and retention effect and blood background signal, presenting an additional source of nonspecific signal (40). This effect is exacerbated not only by the molecular mass of IgG-TAT–based imaging probes but also by the presence of a functional Fc domain, which promotes extended blood uptake through FcRn-mediated recycling (41). In comparison, smaller antibody fragment–based radioimmunoconjugates without a functional Fc domain show a rapid reduction in blood uptake through the renal clearance pathway and lack of FcRn-mediated recycling, potentially reducing nonspecific uptake. Using ^111^In-GFP-G1-TAT–derived smaller antibody fragments may improve sensitivity both in vitro and in vivo. EGFP-targeted imaging is an ideal platform for the evaluation of such optimizations to radioimmunoconjugates.

This work was aimed at guiding research into evaluation of novel intracellular or intranuclear imaging targets. In the context of previous antibody-based PET or SPECT imaging, γH2AX is probably the most documented and has an approximate cell abundance of 800,000 copies per cell at maximum, in an irradiated cell (17), depending on several factors such as cell line and extent of DNA damage before administration of the tracer. Expression levels of proteins we targeted before, such as p21 and p27 levels, may be below the threshold we determined here for GFP. Approximations for these proteins may range from 10^4^ to 10^5^ copies per cell at baseline in normal tissue (27), although few quantitative data are available and levels will oscillate throughout the cell cycle. Although, in this piece of research, we calculated a lower detection limit of approximately 2.4 × 10^5^ copies per cell, we acknowledge that this value is specific to this simplified model system and cannot be treated as an absolute. Therefore, when considering potential candidates for imaging, it is important to take into account the myriad of factors that can influence the lower detection threshold, such as radioimmunoconjugate affinity, CPP selection, and variation in target expression levels and subcellular location. This model system, however, is an endeavor to establish an approximate baseline, and subsequent research will exploit variations of this model to investigate the impact of these factors on the detection capacity of antibody-based or antibody fragment–based intracellular imaging.

## CONCLUSION

This study presents a proof-of-concept methodology set out to evaluate, quantitatively, the practical limitations of antibody-based intranuclear immuno-PET or SPECT imaging. We confirmed the current capacity of imaging an intranuclear epitope with a TAT-conjugated intact antibody. This framework could in the future be used to methodically isolate, interrogate, and optimize a variety of factors that define the efficacy of antibody-based imaging devises, potentially leading to the development of more powerful whole-body immunoimaging devices.

## DISCLOSURE

MRC and CRUK provided financial support through grants MC_ST_OIRO_2019 and 23970, respectively. No other potential conflict of interest relevant to this article was reported

**KEY POINTS**

**QUESTION:** What is the lowest detection threshold for successful imaging of an intranuclear epitope using IgG-TAT–based radioimmunoconjugates?
**PERTINENT FINDINGS:** We developed a model system to test, and improve, the lower target-abundance threshold using IgG-TAT–based PET or SPECT imaging, revealing a current lower detection limit of 180,000 copies per cell in vitro, and 240,000 copies per cell in vivo.
**IMPLICATIONS FOR PATIENT CARE:** This model system can be used to evaluate and improve intranuclear PET or SPECT imaging, with great potential for the development of a range of clinical applications not currently possible.
